# Prevalence and factors associated with diabetic retinopathy among diabetic patients at Arbaminch General Hospital, Ethiopia: Cross sectional study

**DOI:** 10.1371/journal.pone.0171987

**Published:** 2017-03-02

**Authors:** Yilma Chisha, Wondwossen Terefe, Huruy Assefa, Serawit Lakew

**Affiliations:** 1 Department of Biostatistics and health informatics, Arbaminch College of Health Sciences, Arbaminch, Ethiopia; 2 Departement of Biostatistics, Mekelle University, Mekelle, Ethiopia; 3 Department of Maternity and Reproductive Health Nursing, Wolaita Sodo University, Wolaita, Ethiopia; Universita degli Studi di Perugia, ITALY

## Abstract

**Background:**

Currently 93 million people are estimated as living with diabetic retinopathy worldwide. The prevalence and risk factors of diabetic retinopathy in developed countries have been well documented; but in Ethiopia, data on prevalence and associated factors of diabetic retinopathy is lacking.

**Objective:**

To determine prevalence and factors associated with development of diabetic retinopathy among diabetic patients at Arbaminch General Hospital, Ethiopia.

**Method:**

Cross-sectional study design with record review of 400 diabetic patients was conducted at Arbaminch General Hospital from November to January 2015. Among 400 diabetic patients, 270 patients with baseline information and without history of hypertension at baseline were included in this study. But patients with gestational diabetes and with retinopathy at baseline were excluded from the study. Consecutive sampling technique was applied to select study participants. Data of cohorts was extracted from medical record using pre tested structured extraction check list. Data cleaning, coding, categorizing, merging and analysis carried out by STATA version 12. Descriptive statistics was done and presented accordingly. Bivariate binary logistic regression analysis was done to select potential candidates for the full model at P-value cutoff point ≤ 0.25 and multivariable binary logistic regression analysis was made to estimate the independent effect of predictors on the occurrence of diabetic retinopathy. Model diagnostic tests were done, final model fitness was checked using Hosmer and Lemeshow chi square test. Finally, statistical significance was tested at P-value <0.05.

**Result:**

Prevalence of diabetic retinopathy among diabetic patients at Arbaminch General Hospital was 13%. Adjusted analysis showed that the odds of diabetic retinopathy were statistically and significantly associated with baseline age (AOR = 6.06: 95%CI; 2.42, 15.21), baseline systolic blood pressure level (AOR = 4.38: 95%CI; 1.64, 11.68), family history of diabetes (AOR = 0.22: 95%CI; 0.08, 0.58) and duration of diabetes (AOR = 8.84: 95%CI; 3.56, 12.89).

**Conclusion and recommendation:**

In the current study, prevalence of diabetic retinopathy was 13%. Since baseline age ≥60 years, baseline systolic blood pressure level >140 mmhg, duration of diabetes ≥6 years and patients with family history of diabetes were statistically and significantly related with diabetic retinopathy, special care should be given in addition to routine care.

## Introduction

Diabetes mellitus (DM) is a metabolic disorder of carbohydrate, fat and protein and it affects the body’s ability to process and use glucose for energy [1]. Main causes for DM are defects in insulin secretion, insulin action, or both [1, 2]. About 5–10% and 90–95% of patients with diabetes have type1 and type2 respectively [1, 2]. People with diabetes are increasing due to population growth, aging, urbanization and sedentary life style [3]. Since last decade, DM has emerged as an important clinical and public health problem throughout the world and its prevalence reached an epidemic proportion [4, 5].

In 2014, 422 million people in the world had diabetes with prevalence of 8.5% in adult population [6]. A prevalence of diabetes has been steadily increasing for the past three decades and is growing most rapidly in low and middle income countries [4–6]. The epidemic raised in diabetes poses significant public health and socioeconomic challenges through diabetic complications; the eye is the most commonly affected organ by diabetes leading to Diabetic Retinopathy (DR) [7].

There are, 93 million people are approximated as living with DR worldwide [8]. According to the systematic review report, DR affects 7–63% of sub Saharan diabetic patients [9]. Among diabetics In Jimma University tertiary hospital, Ethiopia, prevalence of DR was 25.4% [10]. Prevalence of DR in developed countries has been well documented and risk factors are well known. In study area data on prevalence and associated factors of DR is lacking. This effort constitutes the first attempt to estimate prevalence and associated factors of DR among diabetics at Arbaminch general hospital.

## Methods and materials

This cross sectional study with record review was conducted at Arbaminch general hospital from November to January on diabetic patients ever enrolled since 1990 E.C. Arbaminch hospital was located at Arbaminch town, the capital city of Gamo Gofa zone, which is 505km form Addis Ababa (capital city of Ethiopia) and 280 Km from Hawasa, center of southern nation’s nationality and people regional state (SNNPRS). Arbaminch has two sub cities, Secha and Sikela. The hospital is located at Secha which is the administrative center of Arbaminch town. Though Arbaminch hospital is technically a regional hospital, it is acting as a referral hospital and provides preventive, curative and rehabilitative care for 100,000–200,000 people per year and of them more than 400 patients was diabetic.

### Data collection procedure, sampling and collecting instrument

After taking medical record number of diabetic patients from chronic care follow up clinic, patient folder was drawn from card room by health information technician. Among 400 diabetic patients under follow, 270 patients with baseline information and without history of hypertension at baseline were selected by using consecutive sampling technique. Whereas, Pregnancy induced diabetes and patients with retinopathy at baseline were excluded from the study.

The record reviews were done by two Bachelor of Science (BSc) nurses and facilitated by principal investigators. Training was given for data extractors prior to extraction; Pre test was done on 5% of patients outside study area. Data of cohorts was extracted from medical record by using pre tested structured checklist which was taken from previous studies. After completing data extraction it was transferred to STATA analysis soft ware version 12 for cleaning, coding, categorizing, merging and to check completeness, consistence and outliers.

### Study variables

#### Dependant variable

**Diabetic retinopathy.** The ascertainment of DR status was confirmed by ophthalmologist using history of clinical presentations, visual acuity test result, slit lamp microscope examination and direct ophthalmoscope examination findings.

**Independent variables.** Patient age, Sex, Place of residence was categorized as Urban and Rural. Urban for patients came from Arbaminch town and Rural for patients came from Arbaminch Zuria woreda and other rural Kebeles. Family history of diabetes, level of systolic blood pressure, level of diastolic blood pressure, fasting plasma blood glucose level, duration of diabetes was categorized based on other studies, type of diabetes was categorized as type1 and type2. Type 1 when patient’s age less than 30 years and uses insulin; type 2 when age greater than 30 years and uses oral anti diabetic agents; and type of anti diabetic agents taken are covariates.

### Statistical analysis

After data pre-analysis work was completed, it was analyzed by STATA version 12 and summarized by frequency and percentage to categorical variables and median along with Inter Quartile Range (IQR) to skewed continuous variable. Bi-variate binary logistic regression analysis was carried out to select potential candidate predictors for the full model with cutoff point P-value ≤ 0.25 [11].

Multivariable binary logistic regression analysis was done to estimate the independent effect of predictors on DR.

Model was built and compared by step wise back ward elimination procedure and likely hood ratio test respectively. Interactions and confounders were checked by using change in beta coefficient with cutoff point beta change greater than 20% [11]. Instability of beta-coefficient (multicolinearity) for variables in the final fitted model was checked using variance inflation factor (VIF) with cutoff point mean VIF >10 [12]. Classifying ability (predicting power) of variables in the final fitted model was checked by receiver observed characteristics (ROC) curve and over all goodness of fit was checked using Hosmer and Lemeshow chi square test. Association between predictors and odds of DR was summarized using adjusted odds ratio and statistical significances were tested at p-value <0.05.

### Ethical consideration

This study was conducted after getting ethical approval from Mekelle University College of medicine and health sciences ethical review board, permission to undertake the study was obtained from Gamo Gofa zone health department and Arbaminch general hospital.

## Results

### Socio demographic characteristics of study subjects

From the total study subjects, 220 (81.5%) were within age category <60years. With regard to gender, 138 (51.1%) of study subjects were male. Concerning to place of residence, 150 (56.6%) of study participants were from urban residence. Among 270 study subjects, 231 (85.9%) has no family history of diabetes (Table 1).

**Table 1:** Descriptive statistics with DR status of diabetic patients in Arbaminch general hospital, 2015/16, (n = 270).

Variables	Category	DR status	
		YES	NO
		No (%)	No (%)
Baseline age in year	<60 years	19(52.78)	201(85.9)
	≥ 60years	17(47.22)	33(14.10)
Sex of the patient	Male	21(58.33)	117(50)
	Female	15(41.67)	117(50)
Place of residence	Urban	29(80.56)	121(51.71)
	Rural	7(19.44)	113(48.29)
Family history of DM	Yes	11(14.13)	22(85.87)
	No	25(10.68)	209(89.32)
Type of diabetes	Type1	4(11.11)	66(28.21)
	Type2	32(88.89)	168(71.79)
Duration of diabetes	< 6yrs	15(41.67)	198(84.62)
	≥6 yrs	21(58.33)	36(15.38)
Baseline Systolic BP level	≤140mmhg	24(66.67)	208(88.89)
	>140mmhg	12(33.33)	26(11.11)
Baseline diastolic Bp level	≤90mmhg	30(83.33)	210(89.74)
	>90mmhg	6(16.67)	24(10.26)
Baseline FBG level	176 mg per dl (IQR, 145–253)		
anti DM agents taken	Insulin alone	5(13.89)	73(31.2)
	Insulin and oral	8(22.22)	17(7.26)
	Oral agents alone	23(63.89)	144(61.54)

### Clinical and bio chemical characteristics of study subjects

Thirty six (13%) of study subjects were with DR. Among study subjects, 200(74.1%) were type2 diabetic. Concerning to duration of diabetes, 213(78.9%) were with diabetic duration less than six year. Pertaining towards anti diabetic agents taken, 78 (28.89%) were used insulin alone, 25 (9.26%) were used insulin and oral anti diabetic agents together and the rest 167(61.85%) were used oral anti diabetic agents alone to manage their diabetes. Looking to the baseline systolic and diastolic blood pressure level, 232(85.9%) and 240 (88.9%) of study subjects have baseline systolic and diastolic blood pressure level ≤ 140 mmhg and ≤90mmhg respectively. Fasting plasma glucose level was not normally distributed among cohorts and the median fasting plasma glucose level with inter quartile range (IQR) was 176 (IQR, 145–253) mg per dl (Table 1)

Bi-variate logistic regression analysis was done to select potential candidate variables to the multivariable logistic regression analysis. Based on the pre-set p-value criteria cutoff point ≤0.25(11), except baseline diastolic blood pressure level and sex of the patent, other predictors satisfied the p-value criteria and are a potential candidates for the multivariable logistic regression analysis (Table 2).

**Table 2:** Bi-variate logistic regression analysis of diabetic patients at Arbaminch general hospital, 2015/16, (n = 270).

Variables	DR status		Category	COR	P-value
	YES	NO			
	No (%)	N0 (%)			
**Baseline age in year**	19(52.78)	201(85.9)	<60 years	5.45	0.000
	17(47.22)	33(14.10)	≥ 60years		
**Sex of the patient**	21(58.33)	117(50)	Male	0.71	**0.353**
	15(41.67)	117(50)	Female		
**Place of residence**	29(80.56)	121(51.71)	Urban	0.26	0.002
	7(19.44)	113(48.29)	Rural		
**Family history of DM**	11(14.13)	22(85.87)	Yes	0.20	0.000
	25(10.68)	209(89.32)	No		
**Type of diabetes**	4(11.11)	66(28.21)	Type1	3.14	0.037
	32(88.89)	168(71.79)	Type2		
**Duration of diabetes**	15(41.67)	198(84.62)	< 6yrs	7.70	0.000
	21(58.33)	36(15.38)	≥6 yrs		
**Baseline Systolic BP level**	24(66.67)	208(88.89)	≤140mmhg	4.00	0.001
	12(33.33)	26(11.11)	>140mmhg		
**Baseline diastolic Bp level**	30(83.33)	210(89.74)	≤90mmhg	1.75	**0.260**
	6(16.67)	24(10.26)	>90mmhg		
**Baseline FBG level**	-	-	-	1.001	0.250
**anti DM agents taken**	5(13.89)	73(31.2)	Insulin alone	6.87	0.002
	8(22.22)	17(7.26)	Insulin and oral	2.33	0.099
	23(63.89)	144(61.54)	Oral agents alone		

### Multivariable binary logistic regression analysis

In multivariable binary logistic regression analysis, baseline age, duration of diabetes, baseline systolic BP level and family history of diabetes have statistically significant association with odds of DR.

### Factors influencing diabetic retinopathy among diabetic patients at Arbaminch general hospital

Adjusted binary logistic regression analysis showed that the odds of DR statistically and significantly associated with baseline age of diabetic patients. By holding the effect of baseline systolic BP level, duration of diabetes and family history of diabetes in the final fitted model constant and compare the odds of DR among baseline age < 60 years and ≥ 60 years, odds of DR was six times higher on patients with baseline age ≥ 60 years than their counter parts. (AOR = 6.06: 95%CI; 2.42, 15.21).When baseline systolic blood pressure level increase, odds of DR correspondingly increase. The odds of developing DR was more than four times higher on patients with baseline systolic blood pressure level >140mmhg than their counter parts. (AOR = 4.38: 95%CI; 1.64, 11.68). (Table 3).

**Table 3:** Adjusted binary logistic regression analysis of diabetic patients at Arbaminch general hospital, 2015/16, (n = 270).

Variable	Category	COR	AOR	95%CI of AOR	P-value
Baseline age in year	<60	1	1	2.42	0.000
	≥ 60	5.45	6.06	15.21	
Duration of diabetes in year	<6	1	1	3.56	0.001
	≥6	7.70	8.84	12.89	
Baseline systolic blood pressure level	≤140mmhg	1	1	1.64,	0.003
	>140mmhg	4.00	4.38	11.68	
Family history diabetes	Yes	1	1	0.08	0.002
	No	0.20	0.22	0.58	

**N.B:** 1 is reference category

The odds of DR were high on patients with family history of diabetes than their counter parts; which is 78% less likely on patients without family history of diabetes. (AOR = 0.22: 95%CI; 0.08, 0.58). When duration of diabetes increases, odds of DR also increase. After stabilizing the effect of other covariates in the model constant and compare the odds of DR among patients with duration of diabetes less than six years with greater than or equal to six years, it is nearly nine times higher on patients with diabetic duration greater than or equal to six years than their counters. (AOR = 8.84: 95%CI; 3.56, 12.89) (Table 3).

## Discussion

The current study revealed that prevalence of DR among diabetic patients at Arbaminch general hospital was 13%. This figure is low when compared to study finding in Benghazi Libya, 30.6% of type 2 diabetic patients developed DR [13] and study finding in Jimma University specialized hospital, 33.8% of diabetic patients have visual disturbance [14]. Possible reasons for this inconsistency might be variation on skill and knowledge of physicians to diagnose DR and methods used to detect. It also might be due to variation on advancement of hospitals, number and condition of diabetic patients who served (Jimma university hospital is specialized hospital; hence majority of diabetic cases came by referral after long duration of disease). Furthermore, it might be due to difference in health care system and quality of care given for diabetic patients.

The current study showed that duration of diabetes was directly associated with odds of DR (AOR = 8.84: 95%CI; 3.56, 12.89) (Table 3). The finding is consistent with cross sectional study finding in Jimma university tertiary hospital [14] and longitudinal study finding on type1 diabetic patients in England [15]. Majority of patients with DR in the current study were type2 diabetics and it is consistent with study findings of (13, 14, and 15). This is because type2 diabetics are prone to come late to the health facility because type 2 diabetes is not as severe as type 1 and patients with type 2 diabetes develop DR within short duration of diseases compared to type1[16].

Elevation of systolic blood pressure level is a risk factor for chronic non communicable illness; as well, the current study revealed that baseline systolic BP level was significantly and directly related with the odds of DR (AOR = 4.38: 95%CI; 1.64, 11.68) (Table 3). This finding is in line with longitudinal study finding of United Kingdom [17]. Despite this, the finding of community based cross sectional study in Melbourne, Australia, revealed that, no statistically significant association between level of systolic BP and odds of DR [18]. Possible reason for this irregularity might be variation of patients on self care practice, variation on BP measuring device functionality and skill of health care providers on measuring and recording BP level.

Diabetes affects both individuals and their families and has an impact on economic and social development of a country. Information on availability, cost, and quality of medical care for diabetes does mostly not exist for many low and middle-income countries including Ethiopia. Complications from diabetes, which can be devastating, could largely be prevented by wider use of several inexpensive medicines, simple tests and monitoring and can be a cost saving intervention. This study will provide an in-depth and comprehensive picture of impacts of diabetes and DR and propose clear recommendations for improving prevention and management of diabetic complications. It will help to develop programs and policies for better management of diabetics and cost effective strategies in Ethiopian context.

### Strengths and limitations

#### Strength of the study

Extracted data was recorded in the past at the time when patient came to the health facility, so it was not depended on patient’s memory and it minimized recall bias.

#### Limitations of the study

Since this study was conducted by using pre recorded data, there might be lack of accuracy. Because of institutional based nature of study and non probability sampling technique, findings can’t be generalized for total population. The current study was not used experimental design, so it doesn’t show causal relationship among predictors and DR.

## Conclusion

In conclusion, prevalence of DR was low in the current study when compared with referenced literatures. The study also showed that recommended follow-up examinations and tests for body mass index and blood cholesterol analysis are neither done nor documented for all of patients. The clinic also has no standardized intake form and electronic data base system for diabetic patients, so that some of the most important variables and the effect of life style related factors were not estimated. Finally, baseline age, duration of diabetes, baseline systolic BP level and family history of diabetes were statistically and significantly associated with odds of DR in multivariable binary logistic regression.

## Recommendations

Based on the findings of the current study, the following recommendations are drown for the concerned bodies to overcome the problem and its unwelcomed effects. In addition to routine care, especial emphasis should be given for patients with baseline age ≥60 years, high baseline systolic blood pressure (>140mmhg), patients with duration of diabetes six year and above and patients with family history of diabetes. Knowing and controlling the level of body mass index (BMI), blood cholesterol level and life style related factors were important primary prevention strategies for chronic non communicable illnesses; these factors should be routinely examined, evaluated and recorded. Finally, prospective cohort study is highly recommended to identify real life determinants of DR.

## Supporting information

S1 Dataset (XLSX)
